# Depressive Symptoms Are Associated With Decreased Quality of Life and Work Ability in Currently Working Health Care Workers With Recurrent Low Back Pain

**DOI:** 10.1097/JOM.0000000000002586

**Published:** 2022-06-11

**Authors:** Tarja Virkkunen, Pauliina Husu, Kari Tokola, Jari Parkkari, Markku Kankaanpää

**Affiliations:** From the Department of Rehabilitation and Psychosocial Support, Tampere University Hospital, Tampere, Finland (Dr Virkkunen, Dr Kankaanpää); the UKK Institute for Health Promotion Research, Tampere, Finland (Dr Husu, Mr Tokola, Dr Parkkari); Faculty of Medicine and Health Technology, Tampere University, Tampere, Finland (Dr Virkkunen, Dr Kankaanpää); Faculty of Sports Medicine, University of Jyväskylä, Jyväskylä, Finland (Dr Parkkari).

**Keywords:** depression, pain, nurses, quality of life, work disability

## Abstract

This study investigates the association between depressive symptoms and multisite pain and health-related quality of life and work ability in currently employed health care workers with recurrent low back pain (LBP).

**Methods:**

Multisite pain, depressive symptoms, quality of life, and work ability were assessed by validated questionnaires. A generalized linear model was used for statistical analysis.

**Results:**

Notably, 28% of female health care workers with LBP had at least moderate levels of depressive symptoms. Depressive symptoms were significantly associated with decreased health-related quality of life and work ability. Multisite pain was not significantly associated with work ability.

**Conclusions:**

Depressive symptoms are relatively common in female health care workers with LBP, and treatment of these symptoms may be crucial to improve their work ability.

## Abstract 2

For health care workers, low back pain (LBP) is one of the leading causes of work disability and early exit from the labor market. Moreover, depressive symptoms are common with LBP, and the treatment of these symptoms is a crucial step in improving the quality of life and work ability of health care workers.

## BACKGROUND

Low back pain (LBP) is defined as pain of musculoskeletal origin extending from the lowest rib to the gluteal fold that may at times extend as referred pain into the thigh,^1^ and it is a common and complex global health problem. Moreover, LBP is also a leading cause of disability and results in an enormous economic burden to society. LBP as well as depression were among the three leading causes of the disease burden in terms of years lived with disability in 2017.^2^ Most patients with LBP recover spontaneously, but approximately 10% will go on to develop chronic LBP.^3^ Depression and work-related factors, such as carrying heavy loads at work, and difficult working positions were one the most frequently observed risk factors for chronic LBP.^4^ Estimated 85% to 90% of LBP cases are classified as nonspecific, that is, pain which have not a known pathoanatomical cause.^5^ Nonspecific LBP is associated with lower health-related quality of life (HRQoL), increased functional disability, and increased time off work.^6^ The highest prevalence of LBP has been reported among women aged between 40 and 80 years. LBP frequently causes suffering, an increased number of visits to health care centers, and increased work absenteeism.^7–9^ In addition, long-term musculoskeletal pain is an increasing public health problem that results in significant work absenteeism.^10^ The causes of the high prevalence, incidence, and recurrence of LBP are multifactorial, and LBP should therefore be understood within a biopsychosocial framework.

Typically, health care workers have an increased risk for musculoskeletal disorders, and the most frequently reported disorder is LBP.^11^ Indeed, the 1-year prevalence of LBP among health care workers has been reported to be between 45% and 77%.^12^ In nurses, LBP can lead to impaired quality of life, work disability, and early exit from the labor market.^13^ Occupational risk assessment is a method for estimate health risks from exposure to various levels of a workplace hazard. Furthermore, understanding how much exposure to a hazard poses health risks to workers is important to appropriately eliminate, control, and reduce those risks.^14^ Nurses have several personal, physical, and psychosocial risk factors for musculoskeletal pain that include constrained posture, forceful movement, high emotional strain, and added pressure caused by staff shortages.^15^ Moreover, health care workers often have to work irregular shifts, which frequently leads to sleep disturbances.^16^ Work-related psychosocial factors, such as high job demand, low job control, and low social support, play an important role in the prevalence and incidence of LBP in health care workers. Organizational factors also play an important role in the occurrence of musculoskeletal disorders.^17^ Depressive symptoms were associated with presenteeism independently of pain intensity among health care workers with LBP.^18,19^ Among health care workers, musculoskeletal disorders are an important risk factor for nurses to consider changing jobs or even leaving the nursing profession.^20^

LBP is often concurrent with pain from other body sites.^5^ Multisite musculoskeletal pain has been associated with a greater negative impact on patients’ physical functioning and disability, leading to an increased risk for depressive disorders. Moreover, the reporting of multisite pain appears to worsen the prognosis, as there is an increased likelihood of the problem becoming chronic. Multisite musculoskeletal pain also increases the risk for poor future work ability (FWA).^21^ There is a strong association between number of pain sites and sleep quality as well as psychological distress and overall health.^22^ The aforementioned relationships are complex and interactive and might therefore be components of a larger, multisymptom syndrome. The co-occurrence of musculoskeletal pain and depressive symptoms has a stronger adverse effect on physical work ability and thoughts of early retirement than either one of these alone.^23^

Psychosocial factors play an important role in the development of persistent disabling LBP, and depression might have an adverse effect on the prognosis of LBP.^24^ Depression (major depressive disorder) is a common and serious medical illness that negatively affects how person feels, thinks, and acts. Depression can lead to a variety of emotional and physical problems and can decrease ability to function at work and at home.^25^ Pain and depression often co-occur, share similar symptoms, and may exacerbate each other.^26^ Patients with chronic LBP have significantly impaired psychological status and reduced HRQoL. Comorbidities of depression and chronic pain are highly prevalent in individuals suffering from physical illness.^24^ People with depressive symptoms or diagnosed depression may be at risk for poorer LBP recovery and may require more health care.^27^ Furthermore, the recent study confirmed a relationship between job demands and incident major depression, and this relationship appeared due to neither mediation by nor interaction with LBP.^28^ Depression should be observed in LBP patients to reduce pain-related disability.^29^

Work ability is defined as the physical and mental ability of workers to cope with the demands of their work.^30^ The interactions between the resources of a worker and the demands of the work are intensive and dynamic. Furthermore, these interactions change during aging and the life course. The balance between the human resources of workers, such as health and functional capacities, competence, values, attitudes, and motivation, and work-related factors, such as demands, arrangements, and management, are therefore crucial.^31^ The Work Ability Index (WAI) is a valid, reliable, and commonly used instrument to assess work ability.^30,32,33^ The value of the WAI predicts both register-based disability pension and long-term sickness absence. The WAI consists of two questions: (1) the individual’s own evaluation of work ability compared with lifetime best work ability score (WAS) and (2) the individual’s belief of FWA that predicts both disability pension and long-term (>10 days) sick leave.^34^ The findings of a previous study suggest that the WAS could be used as a simple indicator for assessing the status and progress of work ability among women on long-term sick leave. Furthermore, the predictive value for the degree of sick leave and HRQoL was strong for the WAS.^35^ According to a previous study, the beliefs of workers regarding returning to work, pain intensity, and work strain are predictive of work ability among women on sick leave due to long-term LBP.^36^ Moreover, physical demands in the workplace might cause work disability among workers with LBP.^37^ In addition, individual factors, such as older age, have been associated with poor work ability.^38^ In the general working population, poor work ability combined with one or more chronic diseases is associated with high risk for long-term sickness absence.^39^

The present study investigates the associations between multisite pain and depressive symptoms and HRQoL and work ability in female health care workers with recurrent LBP. Understanding the impact of depressive symptoms and depression is important to clinical management of LBP. Patients with subacute LBP and depression will need psychological care in addition to medical or physiotherapy treatment.^40–42^

## METHODS

### Study Design and Participants

This cross-sectional study was part of a randomized controlled trial (the NURSE-RCT, clinical trial registration NCT01465698) aimed at reducing pain, movement-control impairment, and fear-avoidance beliefs in working female health care workers with recurrent LBP (*n* = 219).^43^ The NURSE-RCT was conducted in the form of three identical consecutive sub-studies. More precise information on the intervention is available in the published article on the protocol of the NURSE-RCT.^43^ The study was approved by the Regional Ethics Committee of Expert Responsibility of Tampere University Hospital, Finland (ETL code R08157).

The inclusion criteria were female sex, aged between 30 and 55 years, worked at current job for at least 12 months, and intensity of LBP at least 2 on the Numeric Rating Scale (scale 0–10) during the past 4 weeks. The exclusion criteria were serious former back injury (fracture, surgery, disc protrusion), chronic LBP defined by a physician or self-report of continuous LBP for 7 months or more, disease or symptoms that limit participation in moderate intensity neuromuscular exercise, regular engagement in neuromuscular-type exercise more than once a week, and pregnant or recently delivered.^43^

## MEASUREMENTS

Questionnaire data were collected during the baseline measurements of the study. Before collection of the baseline measurements, informed written consent was obtained from all participants. The questionnaires used were as follows:

- The modified Finnish version of the 9-item Patient Health Questionnaire (PHQ-9-mFIN)^44^; question items during the past week are as follow: (1) lack of enthusiasm for doing anything, (2) feeling depressed, (3) having trouble getting to sleep or staying asleep, (4) feeling low in energy or slowed down, (5) have a poor appetite, (6) cry easily or feel like crying, (7) feeling bored or having little interest in doing things, (8) feeling lonely, and (9) feeling hopeless about the future.
- The RAND 36-Item Health Survey (RAND-36) with eight subscales^45^: physical functioning, role physical functioning, bodily pain, general health, vitality, social functioning, role emotional functioning, and mental health.
- The short form of the WAI^46^ that is the sum from four questions: current work ability, work ability in relation to physical work demands, work ability in relation to mental work demands, and personal prognosis for work ability in 2 years’ time.
- Multisite pain during the past 4 weeks: number of pain sites three or more (lower back, upper back and neck, shoulders and upper limbs, hips, knees, and lower limbs) with daily or nearly daily pain, and intensity of pain of at least 4 on the scale 0–10.

More precise information on the measurements is given in the study protocol article.^43^

## DEPENDENT (OUTCOME) VARIABLES

The RAND-36, a validated Finnish questionnaire,^45^ was used to assess HRQoL. The RAND-36 includes four physical components (physical functioning, role physical functioning, bodily pain, and general health) and four mental components (vitality, social functioning, role emotional functioning, and mental health). The scores of the eight physical and mental components are then transformed into a scale ranging from 0 to 100. Higher scores represent better quality of life.^45^

Work ability was assessed using the short form of the WAI,^46^ which is the sum score (range, 3–27) from four question items: (1) current work ability (0–10), where 0 = unable to work and 10 = the best possible; (2) work ability in relation to physical work demands (score 1–5; 1 = very poor, 5 = very good); (3) work ability in relation to mental work demands (score 1–5; 1 = very poor, 5 = very good); and (4) personal prognosis for work ability in 2 years’ time (1 = hardly able to work, 4 = not sure, 7 = almost certain work ability).^46^ Current work ability/WAS has been divided into poor (0–7 points) and good (8–10 points).^34^ Personal prognosis for work ability in 2 years’ time/FWA is classified as poor FWA (answers: unlikely or not certain able to work) and good FWA (answer: relatively certain able to work).^34^

## INDEPENDENT VARIABLES

The following were the criteria for multisite pain during the past 4 weeks: (1) number of pain sites ≥3 (lower back, upper back and neck, shoulders and upper limbs, hips, knees, and lower limbs) with daily or nearly daily pain, and intensity of pain of at least 4 on the scale 0–10.

Depressive symptoms were assessed by the PHQ-9-mFIN, which has showed adequate reliability and excellent construct validity among female health care workers with recurrent LBP.^44^ The questionnaire consisted of the following question items during the past week: (1) lack of enthusiasm for doing anything, (2) feeling depressed, (3) having trouble getting to sleep or staying asleep, (4) feeling low in energy or slowed down, (5) have a poor appetite, (6) cry easily or feel like crying, (7) feeling bored or having little interest in doing things, (8) feeling lonely, and (9) feeling hopeless about the future. The scoring of each item was 0 = not at all to 3 = nearly every day leading to a sum score ranging from 0 to 27. Categories of the PHQ-9-mFIN were classified as follow: (1) no depressive symptoms, PHQ-9-mFIN points 0–4; (2) mild depressive symptoms, PHQ-9-mFIN points 5–9; and (3) at least moderate depressive symptoms, PHQ-9-mFIN points 10 or more.^44^

## STATISTICAL ANALYSIS

The associations between the main independent variable and the outcomes were estimated and tested with generalized linear models with gamma distribution due to the skewed distribution of the dependent variables. First, all models included only one independent variable at a time. In the second stage, multiple covariates were included in the models. All nonsignificant covariates (*P* > 0.20) were dropped from the models one at a time. For the parameter estimates and *P* values, 95% confidence intervals were reported for each model. Independent variables with *P* < 0.05 were considered statistically significant. All the analyses were performed using IBM SPSS software (version 24; IBM Corp, Armonk, NY).

## COVARIATES

The covariates included the background variables age, marital status, level of education, and smoking. The work-related factor was shift work, and the health-related factors were perceived health, cardiovascular and respiratory diseases, high blood pressure, and the use of medication. Fitness-related factors included the results of the modified push-up test and 6-minute walk test (6MWT), as well as self-reported physical activity.^43^

## RESULTS

### Descriptive Data of the Study Participants

Characteristics of the study participants are presented in Table 1. The mean age of the participants was 46 years, and they were all female. In total, 41% of the participants were nursing assistants, 47% nurses, and 13% other professionals. The participants had been working for approximately 11 years in their current job. Most of the participants (70%) worked shifts. More than one third (36%) of the participants experienced LBP daily or on most days of the week. The mean LBP intensity was 36 as measured on the 100-mm visual analog scale. The duration of the LBP symptoms was less than 3 months for 65%, 3–6 months for 15%, and more than 6 months for 21% of the study population. In total, 72% of the participants reported multisite pain (musculoskeletal pain at 3–6 sites). The summary score of the depressive symptoms was on average 7.4, indicating mild depression. In total, 28% of participants reported at least moderate depressive symptoms (PHQ-9-mFIN 10 points or more), and 44% reported at least mild depressive symptoms (PHQ-9-mFIN 5–9 points).

**TABLE 1:** Characteristics of the Study Sample (*n* = 219)

Participant Characteristics		Missing
Age, mean (SD), yrs	46.4 (6.8)	
Body mass index, *n* (%), kg/m^2^		
Normal weight (≤24.9)	88 (40.7)	2
Overweight (25.0–29.9)	90 (41.7)	
Obese (≥30.0)	38 (17.6)	
Civil status % single	35.2	
Smoking, *n* (%)		
Nonsmoker	157 (71.7)	
Smoking regularly/occasionally	62 (28.3)	
Profession-related characteristics		
Profession		
% nursing assistants	40.6	
% nurses	46.6	
% other	12.8	
Number of years working in current job, mean (SD)	11.4 (8.8)	2
Shift work, % yes	69.7	1
Subjectively assessed pain characteristics		
LBP intensity (VAS 0–100; past 4 weeks), mean, (SD)	36.2 (22.6)	1
LBP intensity (NRS 0–10), mean (SD)	3.55 (2.1)	1
Frequency of LBP at baseline, *n* (%)		27
Daily	23 (12.0)	
Most days of the week	56 (29.2)	
A few days a week	82 (42.7)	
Recovered from low back pain episodes	31 (16.1)	
Duration of symptoms of LBP at baseline, *n* (%), mo		
<3	140 (64.5)	
3–6	32 (14.7)	
≥6	45 (20.7)	
Number of musculoskeletal pain sites, *n* (%)		2
0–2 pain sites	60 (27.6)	
3 pain sites	68 (31.3)	
4–6 pain sites	89 (41.1)	
Depressive symptoms		
Depressive symptoms* summary score, mean (SD)	7.4 (3.8)	1
No symptoms (score 0–4), *n* (%)	61 (28.0)	
Mild symptoms (score 5–9), *n* (%)	96 (44.0)	
At least moderate symptoms (score 10 or over), *n* (%)	61 (28.0)	

*Depressive symptoms (PHQ-9-mFIN, sum score from 9 questions on the scale 0–27).^32,44^

VAS, visual analog scale.

### Descriptive Data of Health-Related Quality of Life and Work Ability

Descriptive data on the HRQoL are presented in Table 2. The mean physical and mental summary scores of the RAND-36 were 73 and 77 (scale 0–100), respectively. The highest score of the physical components was for physical functioning (85.5), and the highest score of the mental components was for emotional role functioning (84.4). The lowest score of the physical component of the RAND-36 was for bodily pain (63.0), and the lowest score of the mental components was for vitality (63.0).

**TABLE 2:** Descriptive Data for Quality of Life (*n* = 219)

	Study Population	General Population Values*
	Mean (SD)	Mean
	(Missing 1)	
RAND-36 sum score	74.0 (10.0)	
RAND-36 physical component		
Summary score (0–100)	72.9 (15.4)	
Bodily pain	63.0 (19.0)	74.6
Physical functioning	85.4 (13.5)	83.7
Role functioning/physical	74.1 (32.5)	73.5
General health	69.0 (16.5)	65.4
RAND-36 mental component		
Summary score (0–100)	76.8 (16.8)	
Social functioning	83.7 (19.1)	81.7
Vitality	63.0 (18.8)	61.5
Mental health	76.9 (14.6)	73.1
Role functioning/emotional	84.4 (28.4)	73.1

*Finnish population sample of females (*n* = 1133).^30,45^

Descriptive data on work ability are presented in Table 3. The mean WAS of the participants was 7.8 (score 0–10), which reflects moderate work ability. Over one tenth (12%) of the participants were unsure about their FWA after 24 months, which indicates poor FWA. The mean sum of the WAI short form was 22 on a 3–27 scale.

**TABLE 3:** Descriptive Data for Work Ability (*n* = 219)

	Study Population, Mean (SD)	
Work Ability Index, short form (score 3–27)		
Mean (SD)	22.1 (2.6)	Missing 1
Work ability score (score 0–10)		
Mean (SD)	7.8 (1.3)	
Work ability score, classified^a^		
Poor, *n* (%)	77 (35.2)	
Good, *n* (%)	142 (64.8)	
Future work ability, classified^b^		
Poor, *n* (%)	26 (11.9)	
Good, *n* (%)	193 (88.1)	

^a^Work ability score has been classified as poor (0–7 points) and good (8–10 points).^34^

^b^Future work ability has been classified as poor (answers: unlikely or uncertain to be able to work in 2 years’ time) and good (answer: relatively certain to be able to work in 2 years’ time).^34^

## ASSOCIATIONS BETWEEN DEPRESSIVE SYMPTOMS AND MULTISITE PAIN WITH HEALTH-RELATED QUALITY OF LIFE AND WORK ABILITY

According to adjusted analysis regarding the physical subscales of HRQoL, depressive symptoms were only associated with the general health subscale of the RAND-36 (*P* = 0.006) but not with the bodily pain subscale (*P* = 0.426). Furthermore, multisite pain was associated with the general health subscale (*P* = 0.008) and the bodily pain (*P* < 0.001) and physical functioning (*P* = 0.003) subscales of the RAND-36, which indicates that multisite pain was associated with the physical subscales of the HRQoL (Tables 4 and 5).

**TABLE 4:** Associations Between Depressive Symptoms and Health-Related Quality of Life and Work Ability

	Depressive Symptoms			
		95% Confidence Interval		
	B	Lower	Upper	*P*
Health-related quality of life				
RAND-36 physical component				
Bodily pain	0.042	−0.061	0.146	0.426
Physical functioning	−0.011	−0.053	0.032	0.614
Role functioning/physical	0.066	−0.195	0.327	0.620
General health	−0.191	−0.172	−0.029	0.006*
RAND-36 mental component				
Social functioning	−0.194	−0.277	−0.111	<0.001*
Vitality	−0.334	−0.442	−0.225	<0.001*
Mental health	−0.219	−0.281	−0.157	<0.001*
Role functioning/emotional	−0.261	−0.464	−0.058	0.012*
Work ability				
WAI short form	−0.101	−0.172	−0.029	0.006*

*P < 0.05.

Adjustments: bodily pain (shift work [*P* = 0.016], perceived health [*P* < 0.001], hypertension [*P* = 0.022], and 6MWT [*P* = 0.056]); physical functioning (age [*P* < 0.001], perceived health [*P* < 0.001], cardiovascular diseases [*P* = 0.060], hypertension [*P* = 0.011], and 6MWT [*P* = 0.013]); role functioning/physical (perceived health [*P* = 0.014]); general health (perceived health [*P* < 0.001], use of medication [*P* = 0.027], modified push-up test [*P* = 0.123], and 6-minute walk test [*P* = 0.118]); social functioning (perceived health [*P* = 0.087], exercising [*P* = 0.005]); vitality (shift work [*P* = 0.168], smoking [*P* = 0.035], perceived health [*P* = 0.037], modified push-up test [*P* = 0.048]); mental health (civil status [*P* = 0.070], perceived health [*P* = 0.131]); role functioning/emotional (adjustments had no effect); and WAI short form (age [*P* = 0.007], perceived health [*P* < 0.001], modified push-up test [*P* = 0.037]).

**TABLE 5:** Associations Between Multisite Pain and Quality of Life and Work Ability

	Multisite Pain			
		95% Confidence Interval		
	B	Lower	Upper	*P*
Quality of life				
RAND-36 physical component				
Bodily pain	−0.210	−0.310	−0.111	<0.001*
Physical functioning	−0.064	−0.107	−0.022	0.003*
Role functioning/physical	−0.210	−0.463	0.043	0.104
General health	−0.098	−0.171	−0.025	0.008*
RAND-36 mental component				
Social functioning	−0.079	−0.166	0.009	0.077
Vitality	−0.083	−0.208	0.042	0.193
Mental health	−0.052	−0.121	0.017	0.142
Role functioning/emotional	−0.031	−0.251	0.189	0.783
Work ability				
WAI short form	−0.027	−0.064	0.010	0.155

*P < 0.05.

Adjustments: bodily pain (shift work [*P* = 0.007], perceived health [*P* = 0.003], hypertension [*P* = 0.084], 6MWT [*P* = 0.093]); physical functioning (age [*P* < 0.001], perceived health [*P* < 0.001], cardiovascular diseases [*P* = 0.074], hypertension [*P* = 0.017], 6MWT [*P* = 0.014]); role functioning/physical (perceived health [*P* = 0.022]); general health (perceived health [*P* < 0.001], use of medication [*P* = 0.027], modified push-up test [*P* = 0.180], 6MWT [*P* = 0.048]); social functioning (perceived health [*P* = 0.002], exercising [*P* = 0.005]); vitality (shift work [*P* = 0.009], smoking [*P* = 0.023], perceived health [*P* = 0.001], modified push-up test [*P* = 0.054]); mental health (civil status [*P* = 0.033], perceived health [*P* = 0.001]); role functioning/emotional (adjustments had no effect); and WAI short form age ([*P* = 0.047], shift work [*P* = 0.050], perceived health [*P* < 0.001], modified push-up test [*P* = 0.046]).

According to adjusted analysis regarding the mental subscales of HRQoL, depressive symptoms were associated with the social functioning (*P* < 0.001), vitality (*P* < 0.001), mental health (*P* < 0.001), and emotional role functioning (*P* = 0.012) subscales of the RAND-36. However, there was no statistically significant association between multisite pain and any of these (Tables 4 and 5).

According to adjusted analysis, depressive symptoms were associated with the WAI short form (*P* = 0.006). However, there was no statistically significant association between multisite pain and the WAI short form (*P* = 0.155) (Tables 4 and 5).

Non-adjusted associations are presented in Supplementary Tables 1 and 2 (http://links.lww.com/JOM/B142).

## DISCUSSION

In the present study, we found that 28% of all female health care workers with recurrent LBP suffer from at least moderate depressive symptoms. Moreover, the findings of this study indicate that recurrent LBP combined with depressive symptoms has a significant negative impact on mental quality of life and work ability. Contrary to expectations, however, multisite pain was not associated with work ability.

LBP is a leading cause of disability and results in an enormous economic burden.^3^ Health care work is accompanied by several individual, physical, and psychosocial risk factors, and LBP is a common problem among nurses.^11^ In this study, health care workers were still able to work even though they had recurrent LBP. Moreover, most (65%) of the participants had pain symptoms that had lasted for less than 3 months, that is, they had no chronic pain. The reporting of multisite pain appears to worsen the prognosis, and there seems to be an increased likelihood of the problem becoming chronic.^22^ In the current study, as many as 72% of participants reported multisite pain (musculoskeletal pain in three or more sites). Previous studies have concluded that multisite musculoskeletal pain increases the risk for poor future self-perceived work ability.^21^ Furthermore, the co-occurrence of musculoskeletal pain and depressive symptoms has been found to have a stronger adverse effect on physical work ability than either one of these alone.^23^ However, after adjustments, the findings of the present study show that although multisite pain was significantly associated with physical quality of life, it was not associated with mental quality of life or work ability.

Depression might have a significant adverse effect on the prognosis of LBP.^24^ In our study, 28% of participants reported at least moderate depressive symptoms. According to previous studies, people who have depressive symptoms or are diagnosed with depression may be at risk for poorer LBP recovery and may require more health care.^27^ After adjustments, the findings of this study showed that depressive symptoms were significantly associated with decreased mental quality of life and decreased work ability. Against expectations, depressive symptoms were not significantly associated with the bodily pain subscale of the RAND-36.

People with LBP are at risk for poor HRQoL.^6^ The highest score of the physical components of the RAND-36 in the present study was physical functioning, and the highest score of the mental components was emotional role functioning (Table 2). Higher scores represent better quality of life.^45^ The lowest score of the physical component of the RAND-36 was bodily pain, which is lower when compared with the population data for Finnish females of the same age (Table 2).^45^ The lowest score of the mental components of the RAND-36 was vitality 63, which is approximately the same as the population data for Finnish females of the same age.^45^

Among health care workers, musculoskeletal disorders are one important risk factor when considering changing job or even leaving the nursing profession. In addition to leading to an early exit from the labor market, LBP can also lead to work disability in nurses whose work includes lifting, carrying, and other physically demanding tasks.^20^ Previous studies have shown that pain intensity is predictive of self-reported work ability among women on sick leave due to long-term LBP.^36^ Previous studies have also shown that poor self-reported FWA predicts long-term sickness absence, disability pension, and long-term unemployment.^34^ Furthermore, poor self-reported work ability combined with chronic disease is associated with high risk for long-term sickness absence.^39^ In this study, the majority of the participants reported their work ability to be moderate, that is, the mean WAS of the participants was 7.8 (score 0–10). The WAS in these participants was lower than in those members of the Finnish population who were fully able to work.^47^ Furthermore, 12% of the participants were uncertain of their FWA, which can be classified as poor work ability. According to these results, some of these participants might be at risk for long-term sickness absence or even at risk for permanent work disability.

The limitations of the present study include the cross-sectional design and relatively small study sample.

The strengths of the study include the unique study population in terms of non-chronic LBP (ie, recurrent LBP) with physically strenuous work and who were still able to work. Other strengths of the study are the use of the PHQ-9-mFIN, which was recently validated among the present study population,^44^ and the use of the validated questions of the quality of life (RAND-36).^45^

## CONCLUSIONS

The present study found that recurrent LBP combined with multisite musculoskeletal pain or depressive symptoms had a negative association with quality of life among currently working female health care workers. Furthermore, we found that LBP combined with depressive symptoms had a negative impact on work ability. Identifying individuals with a good or unfavorable prognosis among people with LBP is an important goal.^48^ Moreover, recommendations have been made for the use of screening methods in health care to identify those individuals at the early stages of LBP who are at risk for work disability. The aim is to guide them to the appropriate rehabilitation to support their work ability.^49^

Furthermore, the questions relating to depressive symptoms used in this study might be appropriate tools for identifying those health care workers who are at risk for work disability. In addition, assessing the functional capacity of a worker can be used to assess work ability, especially among health care workers who usually have physically and mentally strenuous work. The combination of these estimates could provide more information about a worker’s FWA. Based on the results of the current study, it would be interesting to define the cutoff points for depressive symptoms and multisite pain to ascertain when they begin to significantly affect work ability. Future intervention studies should include psychological aspects alongside physical rehabilitation.
